# Efficacy of azithromycin in sepsis-associated acute respiratory distress syndrome: a retrospective study and propensity score analysis

**DOI:** 10.1186/s40064-016-2866-1

**Published:** 2016-07-28

**Authors:** Kodai Kawamura, Kazuya Ichikado, Makoto Takaki, Yoshihiko Sakata, Yuko Yasuda, Naoki Shingu, Aoi Tanaka, Jyunpei Hisanaga, Yoshitomo Eguchi, Keisuke Anan, Tatuya Nitawaki, Moritaka Suga

**Affiliations:** Division of Respiratory Medicine, Social Welfare Organization Saiseikai Imperial Gift Foundation, Inc., Saiseikai Kumamoto Hospital, Kumamoto, Kumamoto 861-4193 Japan

**Keywords:** ARDS, Azithromycin, Macrolide, Prognosis, Propensity score, Ventilator-free days

## Abstract

**Purpose:**

Acute respiratory distress syndrome is a life-threatening form of respiratory failure without an established pharmacological treatment. Recently, macrolides have been found to be beneficial in cases of acute lung injury, but evidence is limited.

**Materials and methods:**

This single-centre retrospective cohort evaluation of hospitalized patients with sepsis-associated acute respiratory distress syndrome aimed to assess the impact of azithromycin on clinical outcomes by using a propensity score analysis. All data were collected prospectively as part of ongoing research on high-resolution computed tomography of acute respiratory distress syndrome. The primary outcome was 60-day mortality; the secondary outcome was the number of ventilator-free days.

**Results:**

Twenty-nine of 125 patients with sepsis-associated acute respiratory distress syndrome (23.2 %) received azithromycin within 24 h after acute respiratory distress syndrome diagnosis. After adjusting for potentially confounding covariates, azithromycin use was associated with lower 60-day mortality (hazard ratio, 0.31; 95 % confidence interval, 0.11–082; P = 0.02) and a shorter time to successful discontinuation of mechanical ventilation.

**Conclusions:**

Azithromycin use was associated with decreased mortality and ventilator dependency in patients with sepsis-associated acute respiratory distress syndrome. Further well-designed prospective studies are needed.

**Electronic supplementary material:**

The online version of this article (doi:10.1186/s40064-016-2866-1) contains supplementary material, which is available to authorized users.

## Background

Acute respiratory distress syndrome (ARDS) is a life-threatening form of respiratory failure that affects a heterogeneous population of critically ill patients. Although overall mortality appears to be decreasing in recent years because of improvements in supportive care, the mortality rate from ARDS is approximately 30–40 %, and therefore is still high (Rubenfeld et al. 2005; Zambon and Vincent 2008; Force et al. 2012). At present, there is no proven effective pharmacological therapy for ARDS.

Macrolides have immunomodulatory effects in addition to antimicrobial effects. Macrolides have been shown to reduce mortality in many acute lung injury animal models (Tamaoki 2004; Sato et al. 1998; Amado-Rodriguez et al. 2013). In humans, Walkey and Wiener (2012) recently proposed that macrolides may benefit patients with acute lung injury. Walkey reported that the most commonly administered macrolide was erythromycin, followed by azithromycin (40 %) in their registry.

Until 2011, erythromycin was the only macrolide that could be used by intravenous injection in Japan, but erythromycin has many side effects and drug interactions, so we did not routinely use intravenous erythromycin in daily clinical practice. In cases of severe pneumonia, we have used quinolone with beta-lactam antibiotics. Intravenous azithromycin has been approved for clinical use since September 2011 in Japan. Azithromycin is safer and easier to use than erythromycin, and since the publication of Walkey’s report, we routinely use azithromycin for patient with acute respiratory failure from July 2012.

Until now, to our best knowledge, there is no evidence except for Walkey’s report regarding the impact of azithromycin on clinical outcomes in patients with sepsis-associated ARDS.

Therefore, we conducted this retrospective observational study to assess the impact of intravenous azithromycin administration on clinical outcomes in patients with sepsis-associated ARDS by using a propensity score analysis.

## Methods

### Study subjects and design

We performed a retrospective cohort study of patients with ARDS who underwent intensive care during the period from October 2004 to April 2015 at a tertiary academic teaching hospital in Kumamoto, Japan.

This protocol (IRB no. 289) was approved by the Institutional Review Board at Saiseikai Kumamoto Hospital. Written informed consent was obtained from all subjects or surrogates in accordance with the Declaration of Helsinki. If patients were minors, we obtained written informed consent from legal guardians on behalf of the minors.

The primary outcome was 60-day mortality. Additionally, the duration of mechanical ventilation and predictors of 60-day mortality were evaluated.

All data were collected prospectively as a part of ongoing research on high-resolution computed tomography (HRCT) scores of ARDS patients [portions of these results were previously published (Ichikado et al. 2012)]. All patients were directly admitted to the intensive care unit from the emergency department.

The following patients were excluded: those under 15 years of age, those being treated with anticancer drugs or radiation, unsuitable cases such as those involving hematologic disease, and those in the terminal stage of advanced cancer.

Demographics, clinical features, clinical data on admission, and HRCT data were obtained for all patients within 24 h of admission.

ARDS was defined on the basis of the Berlin definition (Force et al. 2012) of ARDS (between 2013 and 2015) and the American–European consensus conference on ARDS (Bernard et al. 1994) (between 2004 and 2012). Patients diagnosed based on the American–European consensus conference definition were re-evaluated to determine whether they met the Berlin definition. All patients were treated using a lung-protective strategy designed by the ARDS Network and according to clinical practical guidelines for acute lung injury and ARDS published by the Japanese Respiratory Society (2010). Sepsis was defined on the basis of the American College of Chest Physicians/Society of Critical Care Medicine criteria (Bone et al. 1992). We divided sepsis into two categories: pulmonary and non-pulmonary.

### Interventions

Before June 2012, there was no patient with sepsis associated ARDS who treated with macrolide. Since July 2012, we use intravenous azithromycin for patients with sepsis associated ARDS within 24 h of ARDS diagnosis. Azithromycin therapy was continued for 5 days if there was no reason to cease administration, such as side effects and arrhythmia, but discontinuation of azithromycin was at the discretion of the attending physician. We did not have specific information about whether patients were treated with oral or intravenous macrolides before admission. No patient was treated with extracorporeal membrane oxygenation therapy in this study cohort.

### Statistical analysis

We summarized baseline characteristics by using percentages for categorical variables and medians and interquartile ranges (IQRs) for continuous variables; comparisons between groups were performed using the Mann–Whitney rank-sum test and Fisher’s exact test, respectively. Survival time in days is reported as the median (95 % confidence interval [CI]), and was calculated from admission until mortality from any cause within the study period. Patients were censored if they were alive at 60 days. Patients who were discharged home before day 60 were considered to be alive at day 60.

Survival curves were plotted using Kaplan–Meier methods. Log-rank tests were used to compare differences in survival. Time to successful discontinuation of mechanical ventilation was evaluated as described previously (Walkey and Wiener 2012).

Adjusted Cox regression modelling was performed using a stepwise selection of variables based on the Akaike information criterion. Variables that may have influenced clinical outcome included age, sex, serum albumin level, serum lactate dehydrogenase level, serum C-reactive protein level, McCabe and Jackson classification, Acute Physiology and Chronic Health Evaluation II (APACHE II) score, PaO_2_/FiO_2_ ratio, white blood cell count, serum antithrombin III level, presence of cirrhosis, presence of pulmonary sepsis, Sequential Organ Failure Assessment (SOFA) score, severity of ARDS based on the Berlin definition, disseminated intravascular coagulation (DIC) score, HRCT score of the lungs at admission, and azithromycin use. The DIC score was based on the DIC scoring system proposed by the Japanese Association for Acute Medicine (Gando et al. 2013). The HRCT score of the lungs was calculated as previously described (Ichikado et al. 2002).

We also estimated adjusted relationships between treatment and outcome using the Cox proportional hazards regression model via inverse probability of treatment weighting (IPTW) using a propensity score. The weights were based on the probability of receiving azithromycin. We used logistic regression models to estimate the propensity to receive azithromycin versus no azithromycin. The model included all variables mentioned above. All tests were two-sided and performed at a significance level of 0.05. We used the R statistical package (version 3.0.1; The R Foundation for Statistical Computing, Vienna, Austria) for all analyses.

## Results

Initially, 185 patients with ARDS were identified, of whom 125 met the criteria for sepsis-associated ARDS. Baseline characteristics are shown in Table 1. Fifty-eight of 125 (46.4 %) patients died within 60 days.

**Table 1:** Baseline characteristics of study

Factor	Group	Overall
		N = 125
Age,y		75 [66, 82]
Sex	M/F	85(68.0)/40 (32.0)
Cirrhosis	No/Yes	112 (89.6)/13(10.4)
Sepsis	Pulmonary	78 (62.4)
	Nonpulmonary	47 (37.6)
APACHE.II		23 [18, 27]
McCabe	1/2/3	114 (91.2)/5(4.0)/6(4.8)
ARDS severity	Mild	10 (8.0)
	Moderate	58 (46.4)
	Severe	57 (45.6)
SOFA		7 [5, 11]
PaO_2_/FiO_2_, mmHg		106.80 [75.1, 139.7]
CT.score		213.3 [184.8, 268.5]
Albumin, g/dL		2.8 [2.40, 3.20]
WBC		9600 [4200, 14,400]
C-reactive protein, mg/dL		15.94 [9.01, 25.70]
Lactate dehydrogenase, IU/L		308 [246, 434]
DIC score		3 [2, 4]
FDP, mg/L		11.10 [1.50, 170.70]
Antithrombin III %		66.00 [52.00, 80.00]
Platelet, X10^4^		17.90 [10.10, 26.40]
D.dimer.μg/ml.		5.60 [2.70, 11.00]
AZM use (%)		29(23.2)
Death (%)		58 (46.4)

n (%), median [IQR]

*APACHE* acute physiology and chronic health evaluation, *ARDS* acute respiratory distress syndrome, *AZM* azithromycin, *DIC* disseminated intravascular coagulation, *FDP* fibrin/fibrinogen degradation products, *SOFA* Sequential Organ Failure Assessment score, *WBC* white blood cell count

**Fig. 1:**
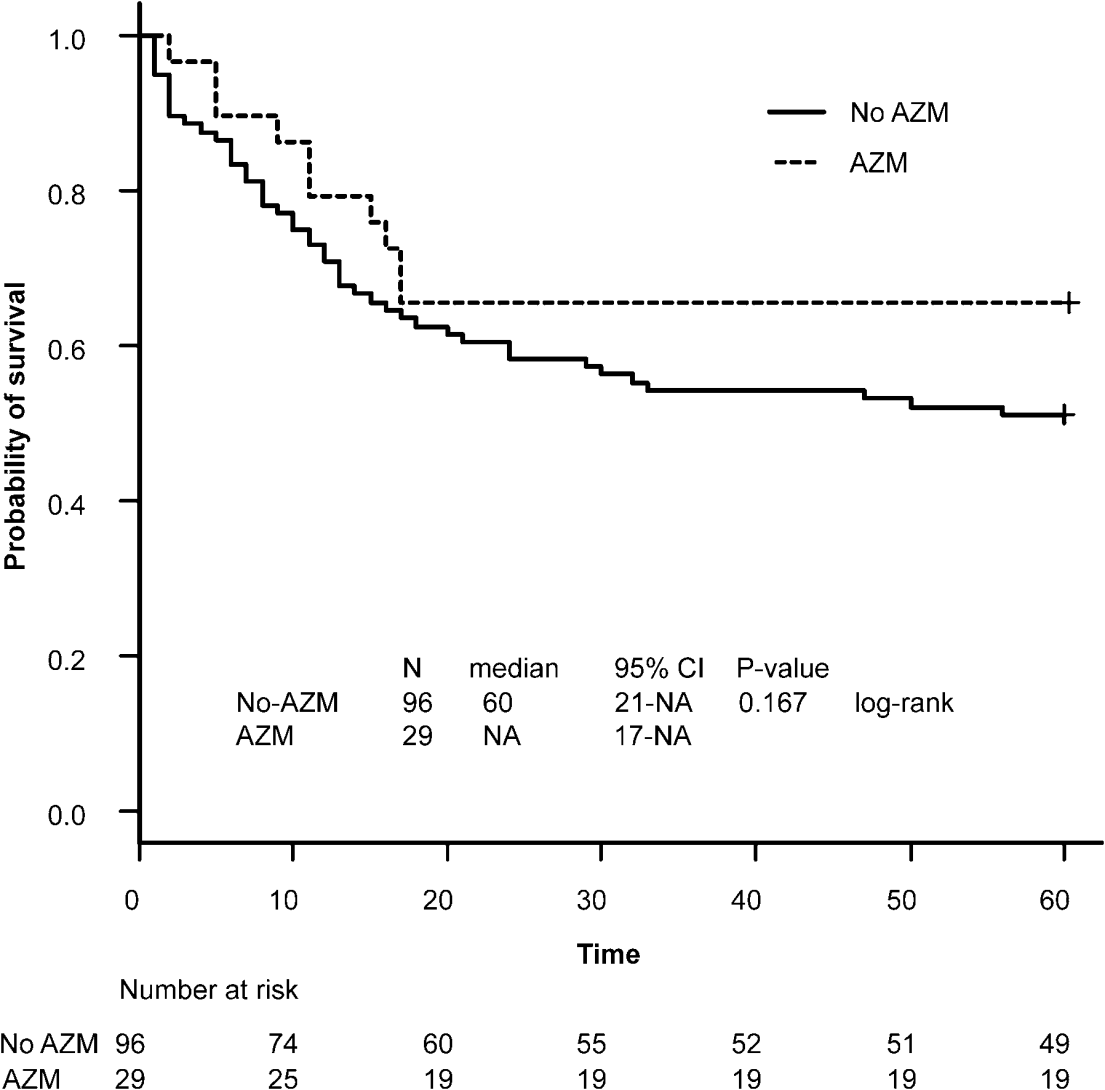
Unadjusted Kaplan–Meier survival curves for patients with acute respiratory distress syndrome treated with and without azithromycin

Twenty-nine of 125 (23.2 %) patients with sepsis-associated ARDS were administered intravenous azithromycin within 24 h of an ARDS diagnosis. The median duration of azithromycin use after ARDS diagnosis was 5 days (IQR, 4–5 days); the mean duration was 4.4 ± 1.0 days. Table 2 shows a comparison of baseline characteristics between patients treated with azithromycin versus those who were not. There were 10 deaths (34.5 %) in the azithromycin group and 48 deaths (50 %) in the control group; the difference in survival was not significant (log-rank test; P = 0.167) (Fig. 1). There were significant differences between the two groups in the cause of ARDS (P = 0.048), PaO_2_/FiO_2_ ratio (P = 0.045), serum albumin level (P = 0.002), DIC score (P = 0.028), and platelet count (P = 0.003).

**Table 2:** Baseline Characteristics of cohort stratified by azithromycin use

Factor	Group	Treatment		P value
		No-AZM	AZM	
N		96	29	
Age (years)		76.5 [67, 82.25]	70 [65, 81]	0.35
Sex	M/F	66 (68.8)/30 (31.2)	19 (65.5)/10 (34.5)	0.821
Cirrhosis	Yes	10 (10.4)	3 (10.3)	1
Sepsis	pulmonary	55 (57.3)	23 (79.3)	0.048
	Nonpulmonary	41 (42.7)	6 (20.7)	
APACHE.II		23 [18, 26]	24 [21, 27]	0.462
McCabe	1/2/3	88 (91.7)/3 (3.1)/5 (5.2)	26 (89.7)/2 (6.9)/1 (3.4)	0.63
ARDS severity	Mild	5 (5.2)	5 (17.2)	0.113
	Moderate	45 (46.9)	13 (44.8)	
	Severe	46 (47.9)	11 (37.9)	
SOFA		7 [5, 11]	9 [7, 12]	0.143
PaO_2_/FiO_2_		100.45 [70.97, 132.70]	114.20 [84.50, 180.00]	0.045
CT.score		214.05 [184.80, 265.60]	211.70 [188.10, 268.50]	0.667
Alb, g/dL		2.90 [2.58, 3.30]	2.50 [2.20, 3.00]	0.002
WBC		10,450 [5250, 14,400]	7300 [2300, 12,200]	0.055
C-reactive protein, mg/dL		15.33 [8.68, 25.07]	19.69 [10.22, 30.10]	0.239
Lactate dehydrogenase, IU/L		312 [248.25, 436.50]	301 [233.00, 434.00]	0.621
DIC score		2 [2, 4]	4 [2, 5]	0.028
FDP, mg/L		11.00 [6.72, 18.10]	11.90 [9.30, 20.00]	0.382
AntithrombinIII, %		66.50 [52.75, 81.25]	65.00 [51.00, 79.00]	0.527
PLT,X10^4^		18.35 [11.93, 28.30]	12.40 [7.70, 18.40]	0.003
D.dimer.μg/ml		5.95 [2.48, 11.00]	4.60 [3.10, 9.30]	0.398
Death (%)		48 (50.0)	10 (34.5)	0.202

n (%), median [IQR]

*APACHE* acute physiology and chronic health evaluation, *ARDS* acute respiratory distress syndrome, *AZM* azithromycin, *DIC* disseminated intravascular coagulation, *FDP* fibrin/fibrinogen degradation products, *SOFA* Sequential Organ Failure Assessment score

**Fig. 2:**
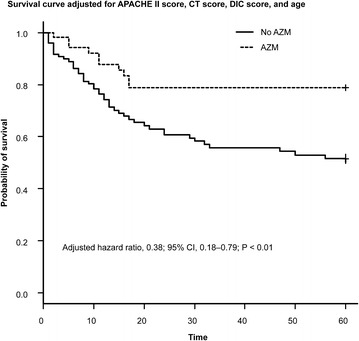
Survival curves for the association between azithromycin use and 60-day mortality from the Cox proportional hazards model, adjusted for age, Acute Physiology and Chronic Health Evaluation II (APACHE II), computed tomography scores, and disseminated intravascular coagulation scores

After adjustment for confounding covariates, azithromycin use was associated with lower mortality (adjusted hazard ratio [HR] 0.38; 95 % CI 0.18–0.79; P = 0.009) (Fig. 2).

In the adjusted analysis, APACHE II score (adjusted HR 1.09; 95 % CI 1.03–1.15; P = 0.004), CT score (adjusted HR 1.22; 95 % CI 1.10–1.35; P < 0.001; HR expressed as mortality change per 10 % increase in area of attenuation with traction bronchiectasis on HRCT), and DIC score (adjusted HR: 1.36; 95 % CI 1.17–1.59; P < 0.001) were also independently associated with a poor prognosis in the regression model (Table 3).

**Table 3:** Cox proportional hazards model results for 60-day mortality

Factor	Hazard.ratio (95 % CI)	P value
APACHE.II	1.09 (1.03–1.15)	0.004
CT.score	1.22^a^ (1.10–1.35)	<0.001
DICscore	1.36 (1.17–1.59)	<0.001
AZM use	0.38 (0.18–0.79)	0.009
Age	1.02 (1.00–1.05)	0.08

*APACHE* acute physiology and chronic health evaluation, *AZM* azithromycin, *CI* confidence interval, *DIC* disseminated intravascular coagulation

^a^Expressed as mortality change per 10 % increase in area of attenuation with traction bronchiectasis on high-resolution CT

IPTW estimators with propensity score adjustment also showed that azithromycin use was associated with lower mortality (HR, 0.31; 95 % CI, 0.11–0.82; P = 0.02).

**Fig. 3:**
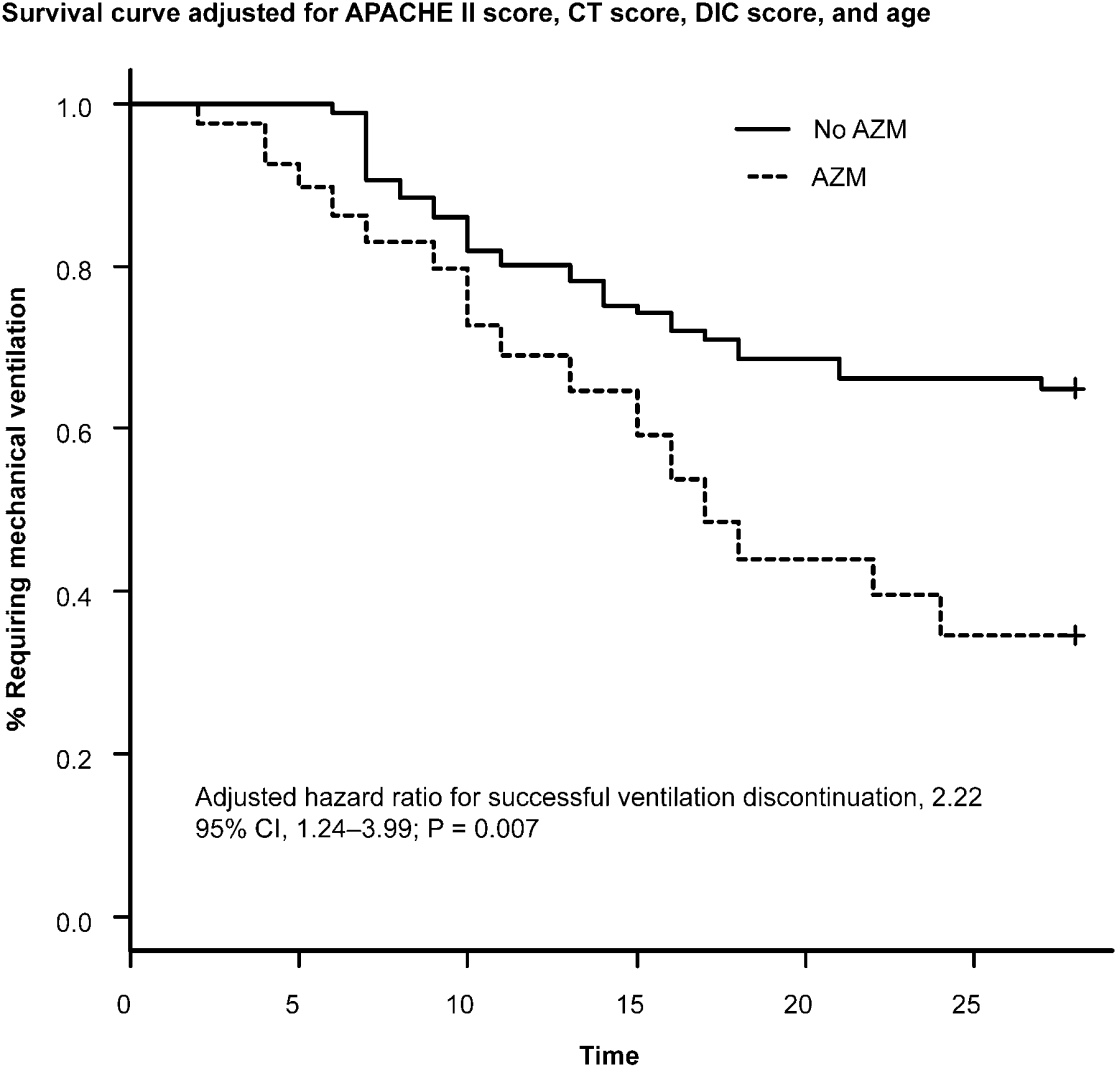
Survival curves for the association between azithromycin use and successful discontinuation of mechanical ventilation at 28 days from the Cox proportional hazards model, adjusted for adjusted for age, Acute Physiology and Chronic Health Evaluation II (APACHE II), computed tomography scores, and disseminated intravascular coagulation scores

 Time to successful discontinuation of mechanical ventilation was shorter in patients treated with azithromycin than in patients who were not (Fig. 3) (adjusted HR for successful ventilation discontinuation: 2.22; 95 % CI 1.24–3.99; P = 0.007).

## Discussion

In this study, we observed lower mortality and a shorter duration of mechanical ventilation use in patients with sepsis-associated ARDS who received intravenous azithromycin within 24 h of sepsis-related ARDS diagnosis. We also identified associations between 60-day mortality, DIC score, and CT score in this cohort.

Azithromycin use within 24 h of diagnosis was associated with improved outcomes in patients with sepsis-associated ARDS. Our result is consistent with Walkey’s report showing that macrolide use was associated with lower 180-day mortality (HR 0.46; 95 % CI 0.23–0.92) (Walkey and Wiener 2012).

There are several reports of macrolide use leading to clinical benefits in patients with infection, especially in severe cases of pneumonia and sepsis (Restrepo et al. 2009; Kumar et al. 2010; Martin-Loeches et al. 2010; Sligl et al. 2014; Garin et al. 2014). Because sepsis is the most common cause of ARDS, macrolide use may lead to improved outcomes in sepsis-associated ARDS, but until now, there has been no evidence to support Walkey’s report. To our knowledge, this is the first report regarding the impact of a macrolide on mortality in patients with ARDS diagnosed by the Berlin definition.

We suggest that the survival benefit of azithromycin in sepsis-associated ARDS is due to anti-inflammatory and immunomodulating effects rather than antimicrobial activity. Walkey and Wiener also proposed that the anti-inflammatory and immunomodulatory effects of macrolides may benefit patients with acute lung injury (Walkey and Wiener 2012). Macrolides have been shown to prevent the production of pro-inflammatory mediators, cytokines, and reactive oxygen species (Kanoh and Rubin 2010), which is potentially the basis for their beneficial effects.

Pneumonia is the most common cause of ARDS. In our cohort, 62.4 % of patients presented pneumonia. The current Infectious Diseases Society of America/American Thoracic Society guidelines for community-acquired pneumonia (CAP) (Mandell et al. 2007) recommend azithromycin use in patients with severe CAP requiring hospitalization on an intensive care unit. Our study supports the current recommendation. Early intervention with azithromycin may suppress excess inflammation that could lead to ARDS and multiple organ failure from severe pneumonia.

There are studies that found that macrolide use did not improve mortality from pneumonia (Asadi et al. 2012; Postma et al. 2015), but these studies included mild and moderate CAP that has low mortality, so a beneficial effect on mortality may not be detected easily. Another study found that macrolide use did not improve mortality in severe infection (Giamarellos-Bourboulis et al. 2014). A recent prospective randomized study assessing the efficacy of clarithromycin in patients with systemic inflammatory response syndrome due to acute pyelonephritis, acute intra-abdominal infection, or primary Gram-negative bacteraemia did not show an advantage in overall mortality (Giamarellos-Bourboulis et al. 2014). However, researchers reported that clarithromycin use shortened the time to resolution of infection, especially in severe sepsis/shock patients, and decreased hospitalization cost. Further, a survival benefit was observed among patients with septic shock and multiple organ dysfunction syndrome in subgroup analyses (Giamarellos-Bourboulis et al. 2014).

Walkey reported that among patients with acute lung injury who were administered a macrolide, erythromycin was the most common (57 %), followed by azithromycin (40 %) (Walkey and Wiener 2012). Azithromycin is the only macrolide that was used in our study population, because azithromycin has fewer drug interactions and fewer side effects than erythromycin, and intravenous clarithromycin is not presently available in Japan. We selected an azithromycin course of 5 days based on a previously reported median duration of macrolide use of 4 days in acute lung injury (Walkey and Wiener 2012). We do not know if a 4–5 day course of a macrolide is long enough to develop favourable effects in patients with ARDS, or if there is a difference in optimal therapy duration between erythromycin and azithromycin, because they have quite different intracellular half-lives; azithromycin has a much longer half-life (40–68 h) than erythromycin. We also do not know whether an oral macrolide improves clinical outcomes in patients with sepsis-associated ARDS because of differing bioavailability.

In this study, we observed that the CT score and DIC score (meaning the presence of DIC) were useful independent prognostic factors for sepsis-associated ARDS. We had reported the utility of a CT score as a prognostic factor in ARDS (Ichikado et al. 2006, 2012), and that result has been reproduced in this study.

The DIC scoring system was proposed by the Japanese Association for Acute Medicine, and it has been reported that it exhibits good prognostic value in predicting multiple organ dysfunction syndrome and a poor prognosis in patients with severe sepsis (Gando et al. 2013). In this study, the DIC score was useful to assess prognosis in patients with sepsis-associated ARDS. Progression from ARDS to multiple organ failure was once thought to be caused by severe ARDS-induced hypoxemia. However, despite advances in respiratory care, many cases progress to multiple organ failure, the onset mechanisms of which are still unknown. Several investigators have previously reported patients with ARDS as having coagulopathy, both systemic and in the alveolar compartment (Bajaj and Tricomi 1999; Ware et al. 2003). Some studies have implicated activation of the coagulation cascade and impaired fibrinolytic activity as pathogeneses of pneumonia and acute lung injury (Gunther et al. 2000) (Schultz et al. 2006). Recently, several investigators have reported on the relationship between coagulopathy and proinflammatory events causing organ injury (Abraham 2000) (Coughlin 2000). Azithromycin has been reported to have immunomodulatory and anti-inflammatory activities, as well as anti-coagulant effects via platelet-activating factor mediated platelet aggregation (Tsoupras et al. 2011). From the viewpoint of coagulopathy in the pathogenesis of ARDS, azithromycin might have contributed to an improvement in survival in sepsis-associated DIC. Further study is needed to clarify the interaction between azithromycin and coagulopathy in ARDS.

Our study has several limitations. First, this study is from a single centre and is retrospective and observational in design. Patients with ARDS who were treated with azithromycin were included in this cohort recently, and there might have been improvements in supportive care over the 10 years that caused differences in outcomes between patients treated with a macrolide and those who were not (Additional file 1). Second, the sample size was small, especially in the azithromycin group, which greatly increases the risk of random error. We adjusted for overt biases by applying IPTW using propensity scores to resolve problems created by unequal chances of receiving treatment. However, we cannot rule out the possibility that unmeasured confounding factors might have affected our results. Because of these potential limitations, the data should be interpreted with caution. Third, the study only involved sepsis-associated ARDS, and therefore this result cannot be applied to ARDS caused by trauma, aspiration, or other factors. Fourth, we do not know valuable information about oral macrolide use before presentation. Fifth, this study did not contain a valuable microbial analysis.

## Conclusions

In conclusion, we observed lower mortality and a shorter duration of mechanical ventilation in patients with sepsis-associated ARDS who received intravenous azithromycin within 24 h of ARDS diagnosis according to the Berlin definition. We also identified associations with 60-day mortality, DIC score, and CT score in this cohort. A further well-designed study is needed to determine whether azithromycin improves morality in sepsis-associated ARDS.

## Additional file

10.1186/s40064-016-2866-1 Trend of 60-day mortality from 2004 to 2015.
