# Supplementary material for: Efficacy of azithromycin in sepsis-associated acute respiratory distress syndrome: a retrospective study and propensity score analysis
Source: Springerplus. 2016 Jul 28;5(1):1193. doi: 10.1186/s40064-016-2866-1 (PMC4963331; doi:10.1186/s40064-016-2866-1)
Supplement: Supplementary file 1 — 10.1186/s40064-016-2866-1 Trend of 60-day mortality from 2004 to 2015. [file 40064_2016_2866_MOESM1_ESM.docx]

Trend of 60-day mortality from 2004 to 2015.
